# New 5,5-(1,4-Phenylenebis(methyleneoxy)diisophthalic Acid Appended Zn(II) and Cd(II) MOFs as Potent Photocatalysts for Nitrophenols

**DOI:** 10.3390/molecules28207180

**Published:** 2023-10-19

**Authors:** Hui-Shi Bin, Hai Hu, Jun Wang, Lu Lu, Mohd Muddassir, Devyani Srivastava, Ratna Chauhan, Yu Wu, Xiaoxiong Wang, Abhinav Kumar

**Affiliations:** 1 School of Chemistry and Environmental Engineering, Sichuan University of Science & Engineering, Zigong 643000, Chinalulusczg@126.com (L.L.);; 2 Department of Chemistry, College of Sciences, King Saud University, Riyadh 11451, Saudi Arabia; muddassir@ksu.edu.sa; 3 Department of Chemistry, Faculty of Science, University of Lucknow, Lucknow 226007, India; devyanisri05@gmail.com; 4 Department of Environmental Science, Savitribai Phule Pune University, Pune 411007, India; 5 School of Materials and Environmental Engineering, Shenzhen Polytechnic University, Shenzhen 518055, China

**Keywords:** MOF, photocatalytic performance, Hirshfeld surface analysis, mechanism

## Abstract

Metal–organic frameworks (MOFs) are peculiar multimodal materials that find photocatalytic applications for the decomposition of lethal molecules present in the wastewater. In this investigation, two new d^10^-configuration-based MOFs, [Zn_2_(L)(H_2_O)(bbi)] (**1**) and [Cd_2_(L)(bbi)] (**2**) (5,5-(1,4-phenylenebis(methyleneoxy)diisophthalic acid (H_2_L) and 1,1′-(1,4-butanediyl)bis(imidazole) (bbi)), have been synthesized and characterized. The MOF **1** displayed a (4,6)-connected (3.4^3^.5^2^)(3^2^.4^4^.5^2^.6^6^.7) network topology, while **2** had a (3,10)-connected network with a Schläfli symbol of (4^10^.5^11^.6^22^.7^2^)(4^3^)_2_. These MOFs have been employed as photocatalysts to photodegrade nitrophenolic compounds, especially *p*-nitrophenol (PNP). The photocatalysis studies reveal that **1** displayed relatively better photocatalytic performance than **2**. Further, the photocatalytic efficacy of **1** has been assessed by altering the initial PNP concentration and photocatalyst dosage, which suggest that at 80 ppm PNP concentration and at its 50 mg concentration the MOF **1** can photo-decompose around 90.01% of PNP in 50 min. Further, radical scavenging experiments reveal that holes present over **1** and ·OH radicals collectively catalyze the photodecomposition of PNP. In addition, utilizing density of states (DOS) calculations and Hirshfeld surface analyses, a plausible photocatalysis mechanism for nitrophenol degradation has been postulated.

## 1. Introduction

Rapid industrialization led to the uncontrolled discharge of varied organic pollutants into water bodies, which has now led to serious environmental pollution [1,2,3]. Amongst different classes of organic pollutants, phenolic compounds comprising nitro substituents, especially *p*-nitrophenol (PNP), are typical organic pollutants existing in industrial waste water. PNP is an essential industrial raw material used in the manufacturing of insecticides, dyes, and pharmaceuticals. However, the USEPA has designated this essential nitrophenolic molecule as a priority pollutant due to its intrinsic toxicity, mutagenicity, and carcinogenicity [4,5,6,7,8]. Therefore, the safe and sustainable eradication of PNP from wastewater discharge is of great concern amongst environmentalists and chemists [4,5,6,7,8].

Currently, to alleviate the concentration of such pollutants in aqueous bodies, several methods such as adsorption, flocculation, membrane filtration, and biological degradation have been applied. However, these methods suffer from several shortcomings and limitations. For instance, adsorption, flocculation, and membrane filtration, which are regarded as physical methods, can merely filter out or collect lethal compounds that always require secondary treatment processes to convert to less lethal substances [9,10,11,12]. Apart from these physical methods, the biological degradation method suffers from its low efficiency and longer response time and elevated cost [12,13,14,15]. Hence, photocatalytic decomposition can be an alternative to minimize the concentration of such organic pollutants in the aqueous medium. However, traditional photocatalysis such as that of TiO_2_ and its composites relies on the use of expensive ultraviolet light sources [13,14,15,16,17]. Nevertheless, because of simple processing techniques, better efficiency, no utilization of secondary treatment processes, lower cost and recyclability [15,16,17,18,19], the visible light photodegradation of such organic pollutants is gaining attention in the chemical science community.

Metal organic frameworks (MOFs) are the assorted sub-class of coordination polymers (CPs) that can be engendered by a rational combination of metal cations, metal clusters with suitable organic ligand and linkers. These materials have showcased potential for their use in a variety of applications because of their large specific surface area and distinctive porous structure [20,21,22,23,24,25,26,27,28,29,30,31,32,33,34,35,36]. In view of their multifarious applications, the design and development of MOFs for their targeted photocatalytic application is gaining attention [37,38,39,40,41]. This is because photocatalytic phenomena operate under light irradiation and can be regarded as low-cost and environmentally safe alternatives [42,43,44,45,46,47,48]. Despite their utility as photocatalysts employed in the decomposition of varied organic compounds, MOFs suffer from some limitations, such as poor framework robustness in aqueous media, inferior recyclability, and the saturation of active sites over the surface [41,42]. Hence, efforts must be devoted to overcoming these limitation associated with MOF-based photocatalysts to improve their photocatalytic performance.

Also, previous reports have suggested that the photocatalytic efficiency of MOFs can be uplifted by using lucidly selected organic ligands [49,50,51]. Hence, varied classes of MOF have been designed and synthesized, employing apt organic linkers, viz., polycarboxylate and N-donor ligands (such as 4,4′-bipy) that exhibit profound photocatalytic performnaces [52,53,54,55]. Hence, in pursuit of new MOFs that can offer efficient photocatalytic properties against the photodegradation of nitrophenolic derivatives, in this investigation, two d^10^-based MOFs comprising Zn(II) and Cd(II) metal cations were synthesized, employing a mixed ligand strategy and selecting the 5,5-(1,4-phenylenebis(methyleneoxy)diisophthalic acid ligand, 1,1′-(1,4-butanediyl)bis(imidazole) (bbi) linker. The relevant findings of these investigations are presented herein.

## 2. Results and Discussion

### 2.1. Molecular Structure Description

#### 2.1.1. [Zn_2_(L)(H_2_O)(bbi)] (**1**)

**Figure 1:**
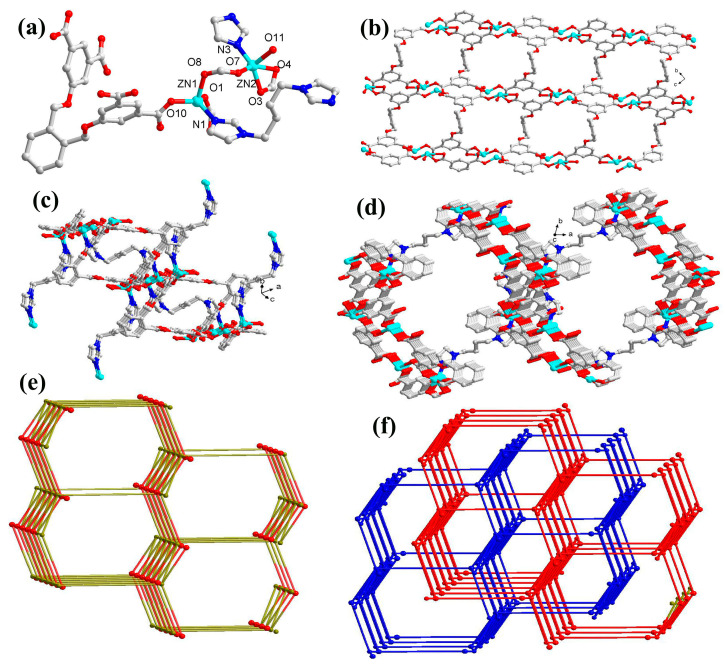
(**a**) Perspective view of the coordination environment around Zn(II) ion in **1**; (**b**) perspective view of the two-dimensional framework in **1** (the bbi ligands have been omitted for clarity); (**c**) perspective view of the bridging mode of bbi in 3D network; (**d**) perspective view of the 3D framework of **1**; (**e**) schematic presentation of the 3D (4,6)-connected network of **1**; and (**f**) perspective view of 2-fold interpenetrating network.

The MOF **1** exhibits a (4,6)-connected 3D framework, wherein the Zn1 center acquires a distorted tetrahedral geometry and is bonded to three carboxylate oxygen centers coming from three L^4−^ ligands (Zn1 − O1 = 1.928(3) Å, Zn1 − O8 = 2.022(3) Å, Zn1 − O10 = 1.925(3) Å) and one bbi nitrogen (Zn1 − N1 = 1.986(3) Å). The Zn2 center possesses a distorted trigonal bipyramidal geometry and is bonded to one bbi nitrogen (Zn2 − N3 = 1.993(4) Å) and three carboxylic oxygen centers from three different L^4−^ ligands and an aqua ligand (Figure 1a). The dihedral angle between the two phenyl rings of the L^4−^ ligand is 47.5° and the four carboxylate groups display dihedral angles of 18.6°, 20.2°, 17.2°, and 21.9° with respect to the plane of corresponding linking aromatic rings. Also, the four carboxylates groups of the L^4−^ ligand exhibit monodentate and bidentate coordination modes and link the Zn(II) ions to engender a 2D layer framework comprising 1D channels (Figure 1b). Furthermore, bbi linkers become entangled in the channels by the Zn−N bond to generate a 3D porous network (Figure 1c,d). Topologically, each Zn2 cluster can be termed as a six-connected node, while each L^4-^ can be considered as a four-connected node and the bbi ligand can act as a linker. Hence, the 3D framework of **1** possesses (4,6)-connected (3.4^3^.5^2^)(3^2^.4^4^.5^2^.6^6^.7) network topology (Figure 1e) and generates a two-fold interpenetrating framework (Figure 1f). Also, the (4,6)-connected framework is microporous in nature, with an 885.5 Å^3^ cavity volume on removal of the solvent molecules.

#### 2.1.2. [Cd_2_(L)(bbi)] (**2**)

**Figure 2:**
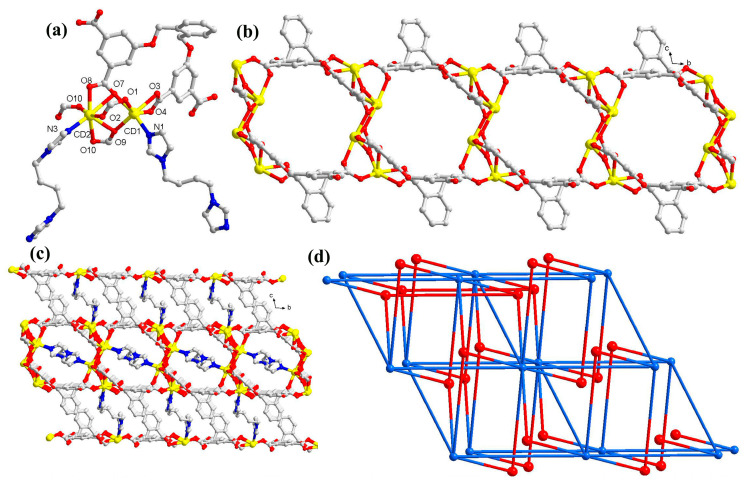
(**a**) Perspective view of the coordination environment around Cd(II) in **2**. All hydrogen atoms are omitted for clarity; (**b**) perspective view of the 2D porous network formed by Cd(II) atoms and L^4−^ ligands; (**c**) perspective view of the 3D framework of **2** along the *bc*-plane with bbi linkers residing in the channels; (**d**) schematic presentation of (3,10)-connected topology.

The MOF **2** crystallizes in a triclinic system with space group *P*-1 and exhibits a (3,10)-connected 3D architecture. In this MOF, the asymmetric unit possesses two crystallographically independent Cd(II) centers, one bptc, one L^4-^ ligand, and one bbi linker (Figure 2a). The Cd1 center adopts a distorted octahedral geometry coordinated to five carboxylate oxygen atoms of four L^4−^ ligands and one bbi nitrogen. The Cd2 center possess pentagonal bipyramidal geometry coordinated to one bbi nitrogen and six carboxylic oxygen centers of bptc. The Cd–O bond lengths are 2.213(4)–2.559(3) Å long, while the Cd–N bond lengths range between 2.223(4) and 2.262(4) Å. The L^4-^ linker connects seven Cd(II) atoms through bidentate bridging (*μ*_2_ − η^1^:η^1^), chelating bridging (*μ*_2_ − η^1^:η^1^), *μ*_2_ − η^1^:η^2^, and *μ*_3_ − η^2^:η^2^ coordination modes for four carboxylate groups to engender the 2D framework (Figure 2b). Further, it forms a 3D framework where L^4-^ linkers are connected in the channels by Cd–N bonds of the bbi linkers (Figure 2c). Topologically, the overall framework of **2** is a (3,10)-connected network with a Schläfli symbol of (4^10^.5^11^.6^22^.7^2^)(4^3^)_2_, with the tetranuclear Cd cluster atoms considered as 10-c nodes and the L^4−^ linker taken as 3-c nodes.

### 2.2. PXRD and TGA

Furthermore, the bulk phase purity values of both MOFs were verified with the help of PXRD measurements (Figures S1 and S2), which indicated close similarities between the experimental and simulated PXRD patterns obtained from the single crystals’ X-ray data. This suggested that both bulk as well as the single crystals of MOFs **1** and **2** crystallized in the same phase.

In addition, thermogravimetric analyses of both **1** and **2** were performed under a nitrogen atmosphere from an ambient temperature to 900 °C at a heating rate of 10 °C/min (Figure S3). In **1**, the first weight loss, occurring between 35 and 98 °C, corresponded to the loss of the coordinated aqua ligand (found 2.1%; calcd. 2.3%). Further, weight loss between 150 and 511 °C could be ascribed to the degradation of the main ligand, suggesting the disruption of the molecular framework.

### 2.3. Diffuse Reflectance Spectroscopy and Photocatalytic Properties

Additionally, UV-Vis spectroscopic experiments were performed to evaluate the optical band gaps of both MOFs that revealed absorption edges at 320 nm. On this basis, the optical band gap energies were calculated to be 3.00 and 3.23 eV for **1** and **2**, respectively (Figures S4 and S5) [56]. Hence, both **1** and **2** possessed semiconducting properties and hence could be utilized as photocatalysts for nitrophenol photodegradation.

**Figure 3:**
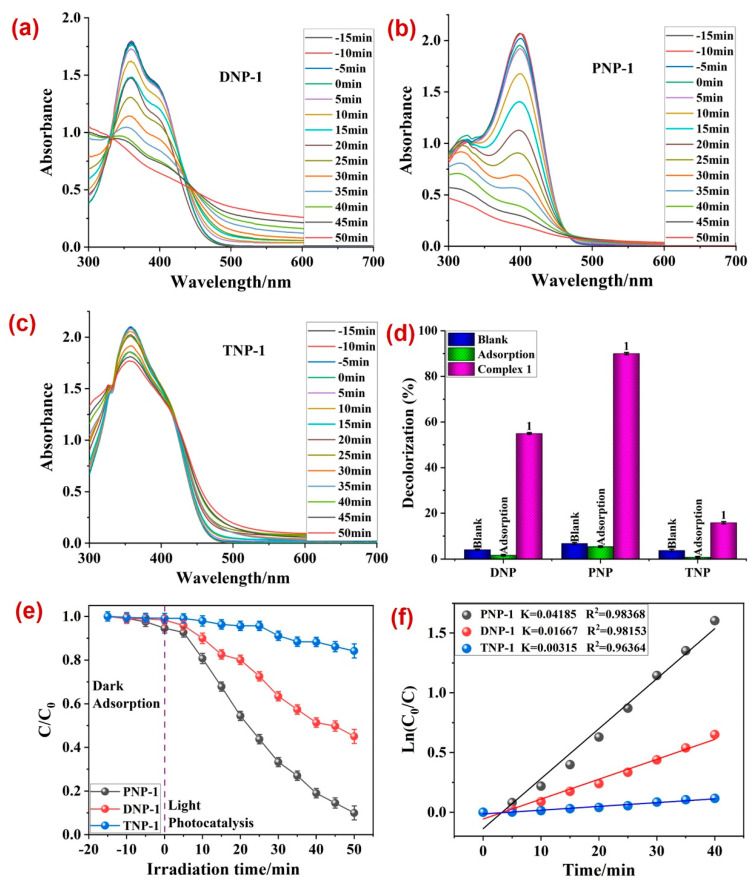
The variation in absorbance of (**a**) DNP, (**b**) PNP, and (**c**) TNP with time in the presence of **1**; (**d**) the percentage photodegradation of DNP, PNP, and TNP in the presence of **1** under different conditions; (**e**) plot between irradiation time and ratio of concentration and initial concentrations; (**f**) rate constants (k) for the photodegradation of nitrophenols.

**Figure 4:**
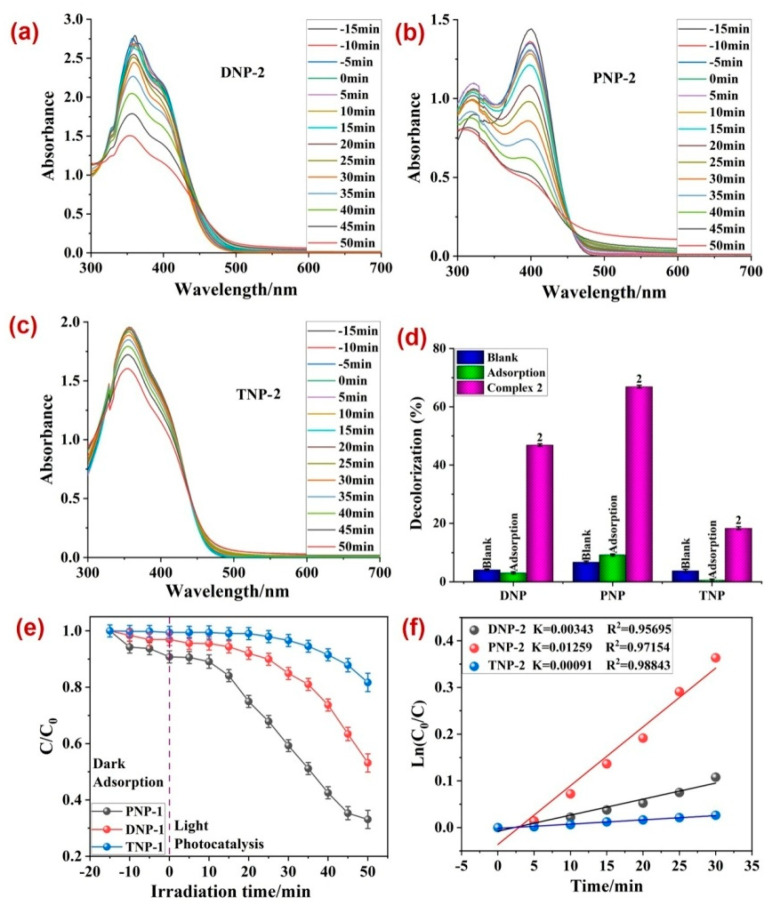
The variation in absorbance of (**a**) DNP, (**b**) PNP, and (**c**) TNP with time in the presence of **2**; (**d**) the percentage photodegradation of DNP, PNP, and TNP in the presence of **2** under different conditions; (**e**) plot between irradiation time and ratio of concentration and initial concentrations; (**f**) rate constants (k) for the photodegradation of nitrophenols.

Hence, both **1** and **2** were utilized as plausible photocatalysts for the degradation of three nitrophenolic compounds, viz., dinitrophenol (DNP), p-nitrophenol (PNP), and trinitrophenol (TNP). The experiments indicated a decline in the characteristic absorption maxima of all three compounds with time (Figure 3a,c and Figure 4a,c) in the presence of both **1** and **2**. Also, amongst all three nitrophenolic compounds, the photodecomposition of PNP by **1** was the highest (~90.01%), in 50 min time (Figure 3d). The MOF **2** also decomposed PNP to the maximum extent, but with a lower percentage decomposition of 68% (Figure 4d). Kinetically, all three photodecompositions followed the pseudo-first-order rate law, with the rate constant (k) for PNP being the maximum, with 0.04185 min^−1^ in the presence of photocatalyst **1** (Figure 3f) and 0.01259 min^−1^ in the presence of photocatalyst **2** (Figure 4f). 

**Figure 5:**
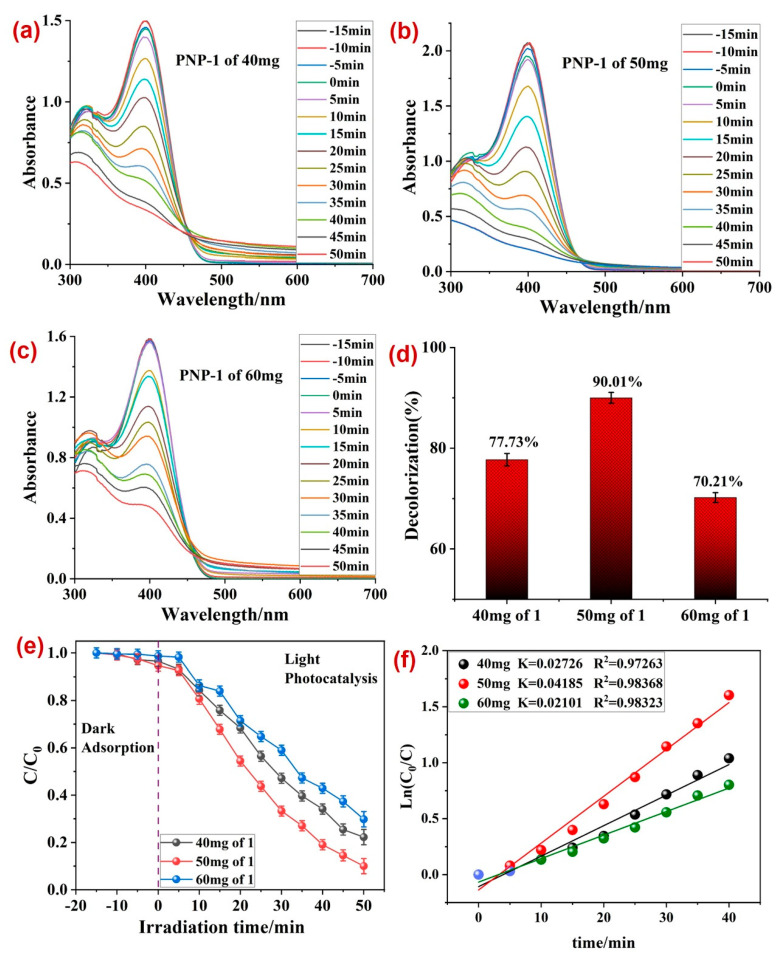
Photodecomposition of PNP at (**a**) 40 mg catalyst dosage, (**b**) 50 mg catalyst dosage, and (**c**) 60 mg catalyst dosage; (**d**) percentage photodecomposition of PNP at different dosages of photocatalyst **1**; (**e**) plot between irradiation time and ratio of concentration and initial concentrations; (**f**) rate constants (k) for the photodegradation of PNP on varying the dosage of **1**. Reaction conditions: PNP (80 mg/L).

Hence, the effect of the photocatalyst dosage and PNP concentration on the photocatalytic performance of **1** was further investigated. In the first experiment, the dosage of photocatalyst **1** varied from 40 mg to 50 mg and 60 mg by keeping the concentration of PNP constant to 80 mg/L (Figure 5a,c). The experiments revealed that a 50 mg dosage of **1** was the optimal photocatalyst dosage at which the maximum photodecomposition of PNP was observed (Figure 5d), with a rate constant k = 0.04185 min^−1^ (Figure 5f). It is worth mentioning here that that elevated photocatalyst dosage (60 mg) exhibited a relatively lower photodegradation of PNP in comparison to 40 and 50 mg dosages. This is because the high photocatalyst dosage blocks the light irradiation that alleviates the photodecomposition of PNP.

**Figure 6:**
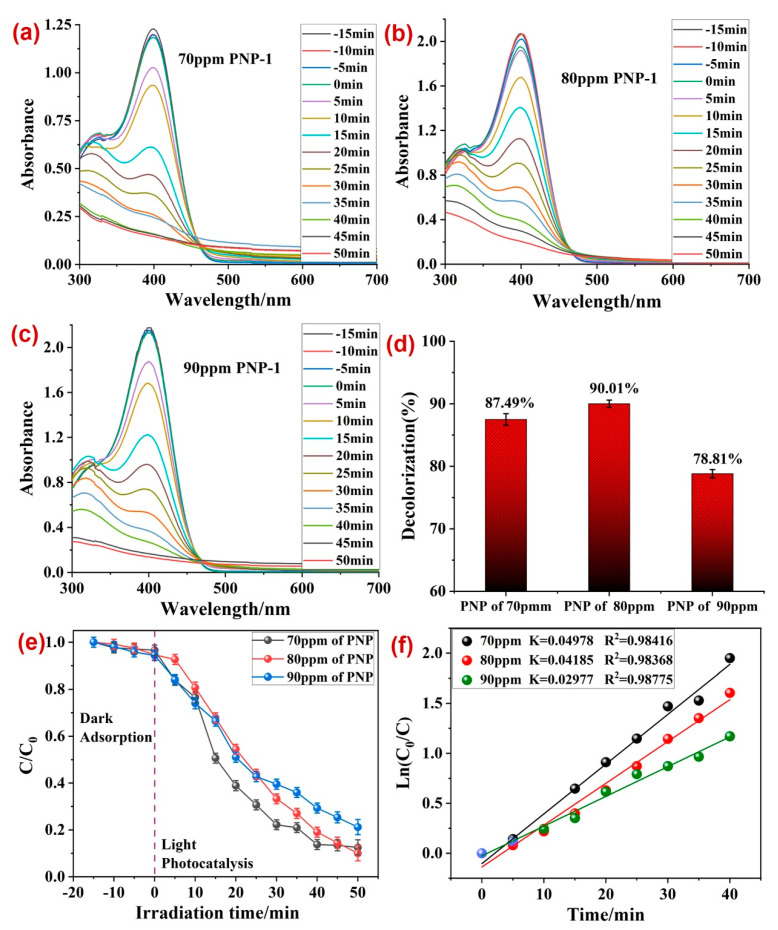
Photodecomposition of PNP by **1** at variable PNP concentrations of (**a**) 70 ppm, (**b**) 80 ppm, and (**c**) 90 ppm; (**d**) percentage photodecomposition of PNP at its variable dosages; (**e**) plot between irradiation time and ratio of concentration and initial concentrations; (**f**) rate constants (k) for the photodecomposition of PNP at varying PNP concentration**.** Reaction conditions: photocatalyst 1 (50 mg).

Also, the initial concentration of PNP may influence its own photodecomposition by the photocatalyst. Figure 6a,c provide an insight into the effect of the initial concentration of PNP (70, 80, and 90 ppm) on its photodecomposition by **1**. The results showed a maximum 90.01% degradation of PNP at its 80 ppm initial concentration with rate constant (k) 0.04185 min^−1^ (Figure 6d). However, at a 90 ppm initial concentration, the photocatalytic efficiency of **1** declined up to 78.81% (Figure 6c,d). This decline in PNP photodecomposition by **1** at a high PNP concentration was because of the surface deposition of PNP molecules/intermediates over photocatalytic materials. This hampered the light absorbing capacity of **1** that decreased its photocatalytic performance. Hence, overall, the best photodecomposition of PNP was observed at a photocatalyst dosage of 50 mg·100 mL^−1^ and 80 ppm PNP concentration, at which 90.01% PNP was found to be photo-decomposed with a rate constant (k) 0.04185 min^−1^.

The pH values’ effect on the degradation of PNP was also explored. The initial pH was conducted at various pHs of 4.0, 6.0, and 8.0 (Figure S10). It was found that the degrading utilization of PNP dramatically reduced at pH 4.0 and 8.0, while it increased highly when the pH value was 6.0. This finding has been reflected by the fact that the positively charged surface of the MOF **1** can benefit the electrostatic interactions with the anionic dyes (Figure S10e) [57,58].

A new 3D three-connected ThSi_2_ (10^3^-b) topological type Ag-MOF of [Ag_2_(ddcba)(4,4-bipy)_2_] constructed from 3,5-(di(2′,5′-dicarboxylphenyl)benozoicacid and 4,4′-bipy shows the photo decomposition with rate constant (k) 0.007 min^−1^ (PNP) at 12 min [59]. A new MOF-based composite with [CoNi(μ_3_-tp)_2_(μ_2_-pyz)_2_] and CuWO_4_ showed a maximum 81% degradation of PNP after 135 min under LED light irradiation [60]. Meanwhile, Fe_3_O_4_/MIL-53(Fe)/H_2_O_2_ was used as a photocatalyst and showed that about 60% of the PNP was photocatalytically degraded after 150 min of visible light irradiation [61]. A new *g*-C_3_N_4_/PDI@MOF heterojunction was synthesized by the in situ growth of NH_2_-MIL-53(Fe) onto the *g*-C_3_N_4_/PDI layer. This material displayed excellent photocatalytic performance for the removal of several water-soluble and toxic organic pollutants (50 ppm) for an efficiency of up to 100% (30 min) under visible light irradiation [62].

**Figure 7:**
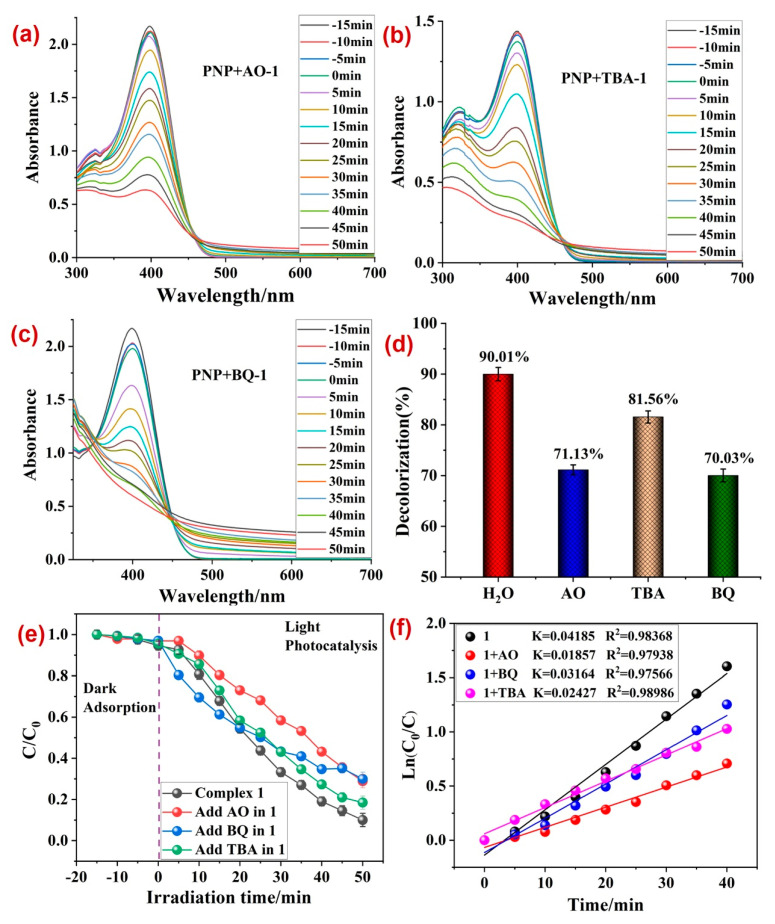
Photodecomposition of PNP in the presence of radical scavengers, (**a**) AO, (**b**) BQ, and (**c**) TBA; (**d**) the percentage photodecomposition of PNP by **1** in the presence of different scavengers; (**e**) plot between irradiation time and ratio of concentration and initial concentrations; (**f**) rate constants (k) for the photodecomposition of PNP in the presence of scavengers. Reaction conditions: PNP (80 ppm); 1 (50 mg).

Additionally, to ascertain the active species that is majorly responsible for the **1** assisted photodegradation of PNP, radical scavenging experiments were performed. To execute these, 10 mg ammonium oxalate (AO, as hole (h^+^) scavenger)/10 mg benzoquinone (BQ, O_2_˙^−^ scavenger)/0.2 mL tertiary butyl alcohol (TBA, ·OH scavenger) were used under the optimized reaction conditions (Figure 7). The outcomes of these experiments indicated that the photodecomposition of PNP declined significantly in the presence of AO and TBA, which are hole and ·OH scavengers, respectively (Figure 7a,b), as evident in the decline in the kinetic rate constant (k) in the presence of AO and TBA from 0.04185 min^−1^ to 0.01857 and 0.02427 min^−1^, respectively. Hence, it can be concluded that both holes and ˙OH radicals are the main reactive species responsible for the **1**-assisted photodegradation of PNP [57,58,59,60,61,62].

**Figure 8:**
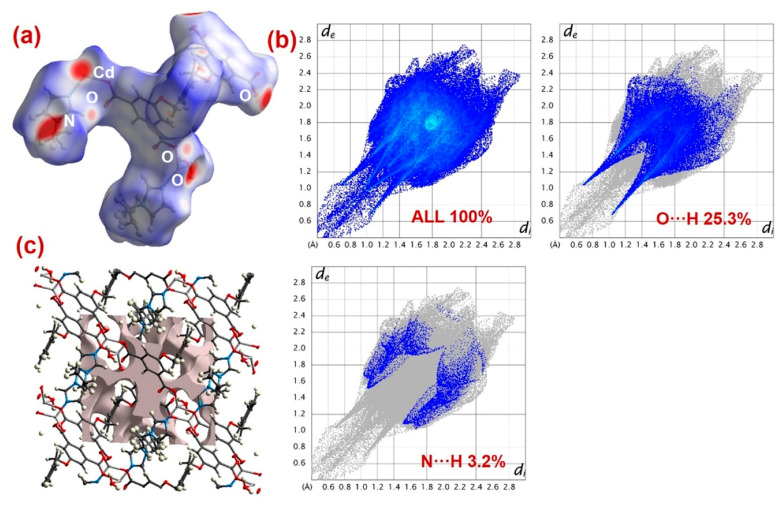
(**a**) *d*_norm_, (**b**) total and decomposed fingerprint plots, and (**c**) outcome of void volume calculations for **1**.

**Figure 9:**
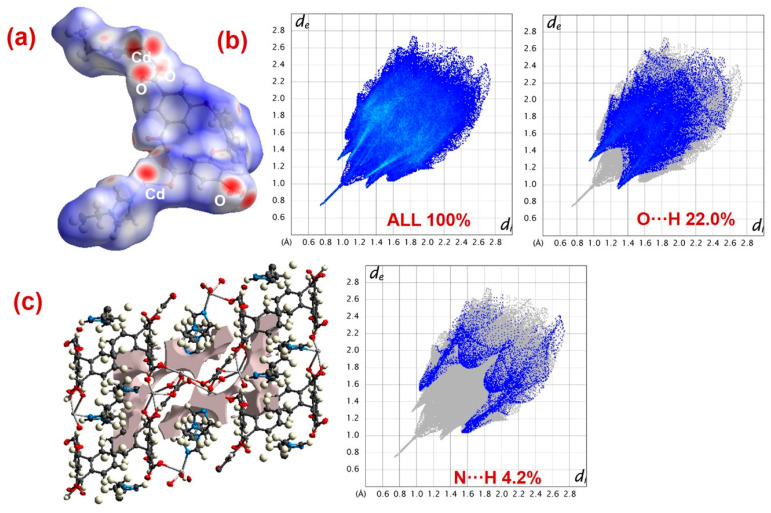
(**a**) *d*_norm_, (**b**) total and decomposed fingerprint plots, and (**c**) outcome of void volume calculations for **2**.

Hirshfeld surface analyses were executed to address weak interactions existing in MOFs **1** and **2** (Figure 8 and Figure 9) [63,64,65,66,67]. A very pertinent outcome of this analysis is the *d*_norm_ plots that were drawn in the range of −0.5 to 1.5 for both the MOFs (Figure 8a and Figure 9a). The *d*_norm_ surfaces for both **1** and **2** displayed intense red spots that indicated strong interaction regions. At these regions of the MOFs, PNP and analogous nitrophenol molecules may undergo interactions to undergo photodecomposition [63,64,65,66,67]. Also, the fingerprint plots for both **1** and **2** suggested that both MOFs could form O···H and N···H interactions, which enabled the interaction of PNP molecules at the MOF’s surface (Figure 8b and Figure 9b). However, in both the MOFs, as compared to N···H interactions, the percentage contribution of O···H interactions was relatively larger.

Additionally, using the common void cluster parameter “unit cell + 5.0 Å,” the crystal lattice void volumes for both **1** and **2** were calculated (Figure 8c and Figure 9c) [68,69]. The outcomes suggested that **1** had a lattice void volume of 208.79 Å^3^ and a void area of 609.40 Å^2^, while **2** had a lattice void volume of 191.60 Å^3^ and a void area of 552.49 Å^2^. These parameters suggested the microporous nature of both the MOFs, with void spaces where PNP molecules could interact and become decomposed. Also, the better photocatalytic efficiency of MOF **1** could be attributed to its relatively better O···H interaction contribution, as well as its greater void volume and void area than **2**.

**Figure 10:**
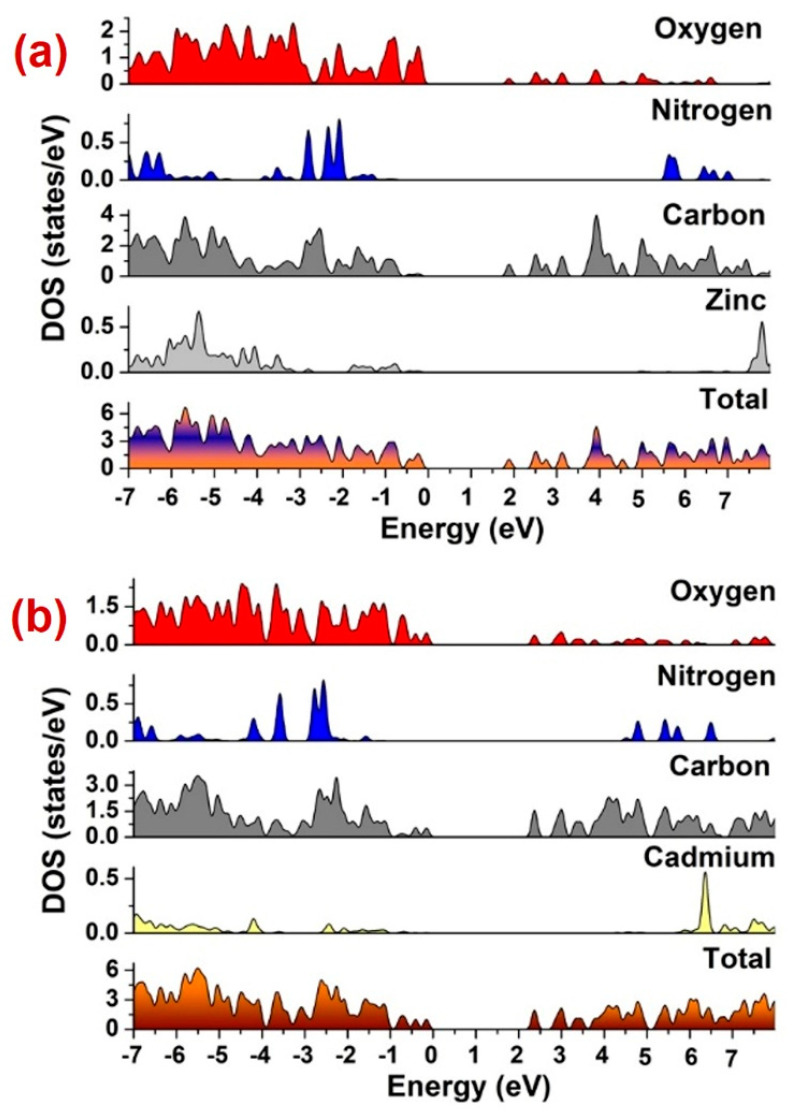
Density and partial density of states plots for (**a**) **1** and (**b**) **2**.

Further, band gap calculations were performed to assess the plausible photocatalytic mechanism for the MOF-aided PNP photodegradation (Figure 10). The outcomes of these calculations indicated that in both **1** and **2**, aromatic carbon and carboxylate oxygen centers contributed to the valence band. Also, in **1**, a small contribution from the Zn(II) center was also evident. Also, the conduction bands between 1.8 and 2.0 eV in **1** and 2.2 and 2.5 eV in **2** had contributions from aromatic carbon and oxygen centers. Hence, these calculations suggest that the main electronic transitions in both **1** and **2** were intraligand and ligand-to-ligand, with an admixture of metal orbitals in **1**. These transitions are primarily responsible for photocatalysis. Apart from this, the MOF **1** possessed a relatively smaller band gap than that of **2**, which is the other reason for its better photocatalytic efficacy compared to **2**.

**Scheme 1:**
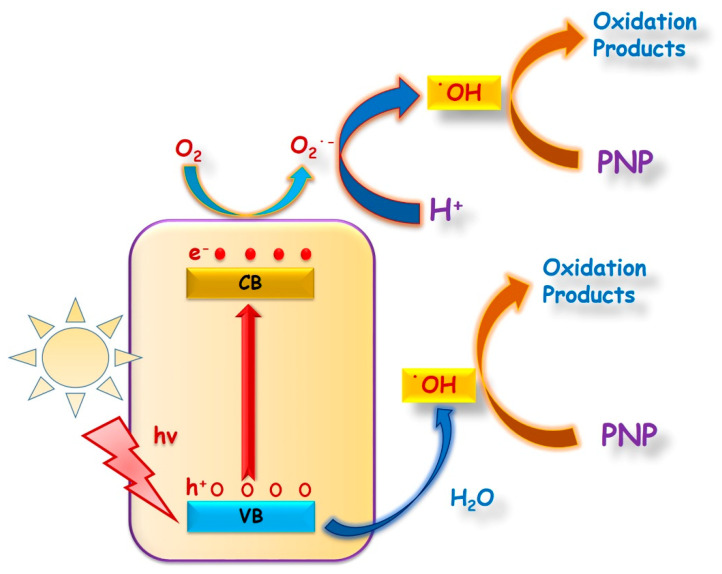
Plausible photocatalytic mechanism.

Hence, the overall photocatalytic mechanistic pathway might be operating as follows: (1) In presence of light, the electrons of MOFs are excited from VB→CB to generate equivalent numbers of holes (h^+^) in VB. The hydroxyl radicals (•OH) are then generated by the reaction of H^+^ with O_2_⁻. The O_2_⁻ are generated by the reduction of O_2_ by e⁻ and the oxidation of OH by holes (h^+^) (Scheme 1).

Further, the recyclability and reuse of any photocatalyst is very important. Hence, after the photocatalytic decomposition of PNP, the MOFs **1** and **2** are isolated from the reaction mixture, washed repeatedly with the solvent, and reused again for the fresh catalytic cycle (Figures S6 and S7). The recycle experiments revealed no perceptible change in the photocatalytic performance of **1**, even after four catalytic cycles. Further, the phase purity and structural robustness after photocatalysis was assessed with the aid of the PXRD measurement for the recovered photocatalyst **1** (Figures S1 and S10), which indicated no alteration in the peak positions in the PXRD plot of the recycled **1** with respect to the PXRD pattern of the pristine sample of **1**. This indicates that **1** preserved its phase purity and structural integrity after photocatalytic experiments. Further, scanning electron microscopy (SEM) was also performed for both **1** and **2** before and after photocatalysis (Figure S8). The pre- (Figure S8a,c) and post-catalysis (Figure S8b,d) SEM imaging revealed no major changes in the morphologies of either of the MOFs, which did not change significantly after the photocatalysis, thereby showing the material robustness of the MOFs. 

## 3. Conclusions

In this study, two new d^10^-configuration-based Zn(II) and Cd(II) MOFs were synthesized using the 5,5-(1,4-phenylenebis(methyleneoxy)diisophthalic acid ligand and 1,1′-(1,4-butanediyl)bis(imidazole) (bbi) linker. The Zn(II) MOF displayed a (4,6)-connected (3.4^3^.5^2^)(3^2^.4^4^.5^2^.6^6^.7) network topology, while Cd(II) had a (3,10)-connected network with a Schläfli symbol of (4^10^.5^11^.6^22^.7^2^)(4^3^)_2_. These newly synthesized MOFs can be used as photocatalysts to photodegrade nitrophenolic compounds, especially *p*-nitrophenol. Amongst both the MOFs, the Zn(II) offered relatively better photocatalytic performance, which was attributed to relatively better O···H interaction contribution as well as a greater void volume and void area than the Cd-based MOF. Apart from this, the relatively smaller band gap in Zn-based MOF in comparison to the Cd-based MOF might be another reason for the better photocatalytic performance. Further, the photocatalytic efficacy of the MOF was assessed by altering the PNP concentration and photocatalyst dosage, which indicated that under the optimal reaction condition, viz., 80 ppm PNP concentration and 50 mg photocatalyst concentration, an ~90.01% photodegradation of PNP was observed in a 50 min time span. Therefore, it can be concluded that the use of mixed ligands can result in the development of novel d^10^-configuration-based MOFs with tuned electronic properties that not only have structural robustness, but also show desirable photocatalytic properties for the safe and long-term degradation of PNP and related nitrophenols for real water samples that contain such pollutants. 

## 4. Experimental Section

### 4.1. Materials and Methods

Comprehensive descriptions of the methods and equipment used in the study presented are included in the electronic supporting material (ESI).

### 4.2. Synthesis of ***1***

A homogenous solution of H_4_L (0.15 mmol, 0.070 g), bbi (0.25 mmol, 0.074 g), Zn(NO)_3_·6H_2_O (0.40 mmol, 0.119 g), 10 mL of H_2_O, and 2 drops of HNO_3_/H_2_O (*v/v* = 1:1) was prepared and after stirring for 30 min this was thereafter transferred into a 25 mL Teflon-lined reactor, sealed and heated to 180 °C for three days and then cooled with a cooling rate of 5 °C/h to give a colorless block-like crystal of **1** with 72% yield based on zinc. IR (cm^−1^): 3412 vs, 1678 s, 1611 s, 1570 s, 1457 s, 1412 m, 1091 m, 758 m. See the ESI (Figure S11).

### 4.3. Synthesis of ***2***

A homogenous solution of H_4_L (0.15 mmol, 0.070 g), bbi (0.25 mmol, 0.074 g), Cd(NO)_3_·4H_2_O (0.40 mmol, 0.123 g), 10 mL of H_2_O, and 2 drops of HNO_3_/H_2_O (*v/v* = 1:1) was prepared and, after stirring for 30 min, this was transferred to a 25 mL Teflon-lined reactor, sealed and heated to 180 °C for three days and then cooled with a cooling rate of 5 °C/h to give a yellow block crystal of **2** with 63% yield based on cadmium. IR (cm^−1^): 3012 vs, 1640 s, 1605 s, 1571 s, 1422 s, 1398 m, 1081 m, 771 m. See the ESI (Figure S11).

### 4.4. Photocatalysis 

In a typical process, 50 mg of MOF **1** or **2** was added to 60 mL aqueous solution of nitrophenols and stirred for half an hour in the dark to achieve an adsorption equilibrium between MOF and nitrophenols, and then it was irradiated using a 350 W Xe lamp. About 2 mL of this suspension was isolated after every 10 min and centrifuged and UV-Vis spectroscopies for the obtained solutions were performed using a Varian 50 UV/Vis spectrophotometer. The photograph of the photocatalytic setup is shown in Figure S13.

### 4.5. Computational Details

Theoretical calculations were used to elucidate the putative photocatalysis mechanism, and for this a unit of MOF was geometrically optimized using the B3LYP function [70] employing the 6–31G** basis set for all atoms except Zn and Cd, for which the CEP-121G basis set was used. The final coordinates of both MOFs were employed for band structure calculations using periodic boundary calculations (PBC), employing the Gaussian 09 program [71]. The density of state (DOS) and partial DOS plots were constructed using GaussSum 3.1 [72].

### 4.6. Hirshfeld Surface Analyses

Molecular Hirshfeld surfaces analyses were performed using previously reported protocols [73,74].

## Data Availability

Not applicable.

## Supplementary Materials

The following supporting information can be downloaded at: https://www.mdpi.com/article/10.3390/molecules28207180/s1, Figure S1: The PXRD plots for **1**; Figure S2: The PXRD plots for **2**; Figure S3: The TGA plots for **1** and **2**; Figure S4: Solid state UV-Vis spectra for 1 and 2; Figure S5: The optical band gap plots for 1 and 2; Figure S6: The photocatalytic recycle experiments for the photodegradation of PNP using **1**; Figure S7: The photocatalytic recycle experiments for the photodegradation of PNP using **2**; Figure S8. SEM images for 1 (a) before catalysis and (b) after catalysis and SEM images for 2 (c) before catalysis and (d) after catalysis; Figure S9: The PXRD plots for **2** that after photocatalysis; Figure S10: (a–c) the photocatalytic efficacy under various pH values; (d) the relationship of the line for C_0_/C and t under different pH; (e) the comparison efficiency under various pH values; Figure S11: View of the IR for MOFs **1** and **2**; Figure S12: Adsorption-desorption isotherms of the as-synthesized samples in this work (the pore size distribution curves (insert)); Figure S13: Photograph of the photocatalytic setup in this system; Table S1: Crystallographic data and structure refinement details for **1**–**2**; Table S2: Selected bond distances (Å) and angles (deg) for **1**–**2**; Table S3: Hydrogen Bonds Distances (Å) and angles (deg) for **1**–**2**.
