# Supplementary material for: New 5,5-(1,4-Phenylenebis(methyleneoxy)diisophthalic Acid Appended Zn(II) and Cd(II) MOFs as Potent Photocatalysts for Nitrophenols
Source: Molecules. 2023 Oct 19;28(20):7180. doi: 10.3390/molecules28207180 (PMC10608887; doi:10.3390/molecules28207180)
Supplement: Supplementary file 1 [file molecules-28-07180-s001.zip › molecules-2616493-supplementary.pdf]

New 5,5-(1,4-phenylenebis(methyleneoxy)diisophthalic acid appended Zn(II) and Cd(II) MOFs as potent photocatalysts for nitrophenols

X-ray crystallographic data collection and structural determination

The crystallographic diffraction data for **1–2** were collected on a Bruker SMART APEX II CCD diffractometer with graphite-monochromated Mo Ka radiation (k = 0.71073 Å) at 296(2) K using the u/x scanning technique. All the structures were solved using direct methods and successive Fourier difference synthesis, and refined using the full-matrix least-squares method on F^2 with anisotropic thermal parameters for all non-hydrogen atoms by SHELXS-97. An empirical absorption correction was applied using the SADABS program. Basic information pertaining to crystal parameters and structure refinements is summarized in Table S1. Selected bond lengths and angles for 1–2 are listed in Table S2. Hydrogen bonding distances and angle data are listed in Table S3, shown in the Supplementary data. CCDC: 2282867- 2282868.

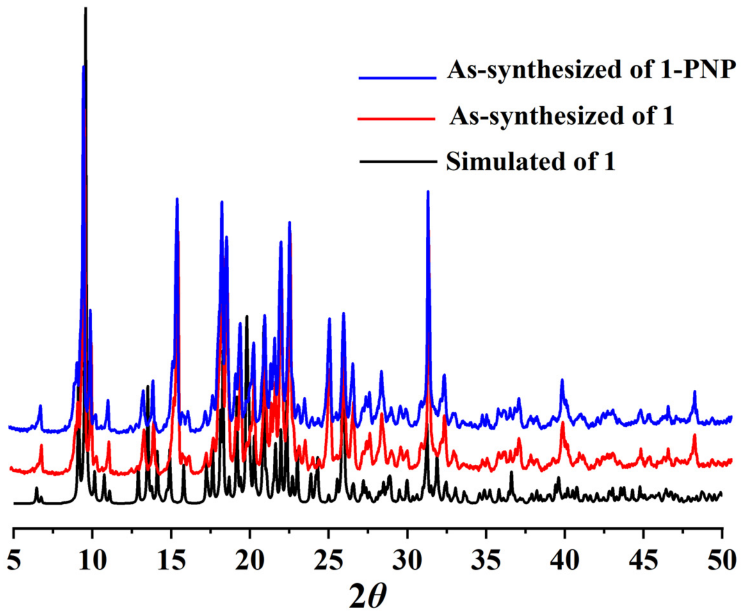

Figure S1 The PXRD plots for **1**.

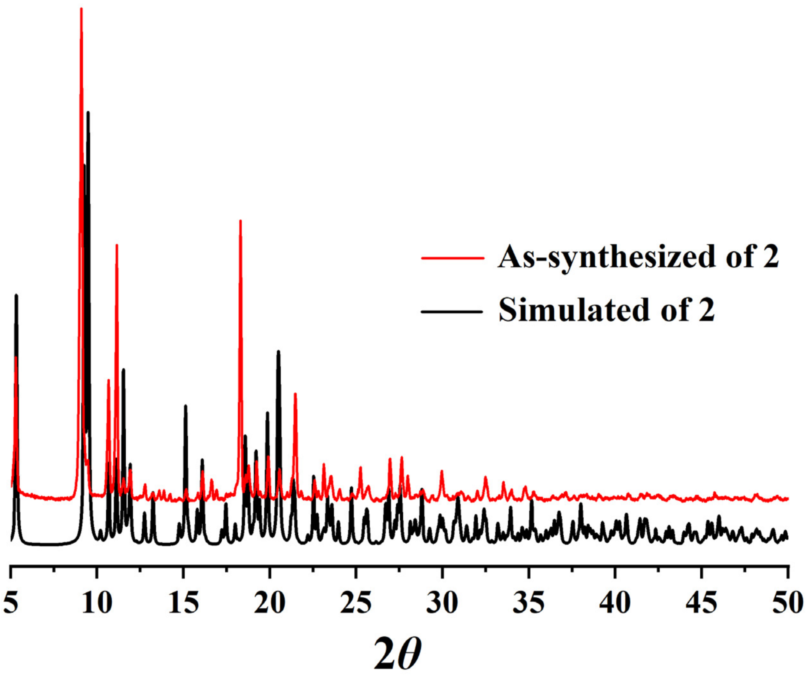

Figure S2 The PXRD plots for **2**.

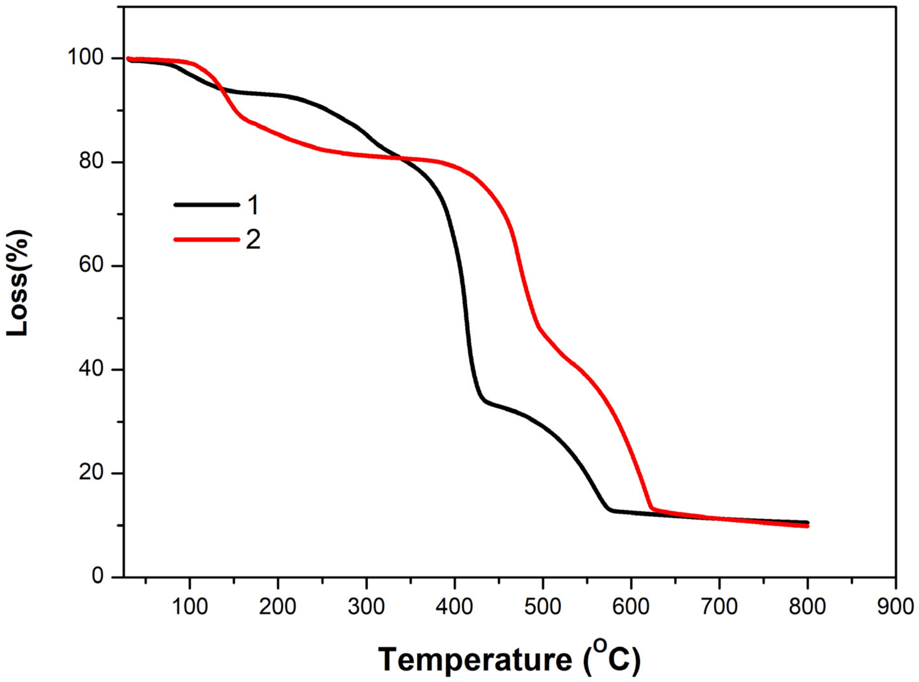

Figure S3 The TGA plots for **1** and **2**.

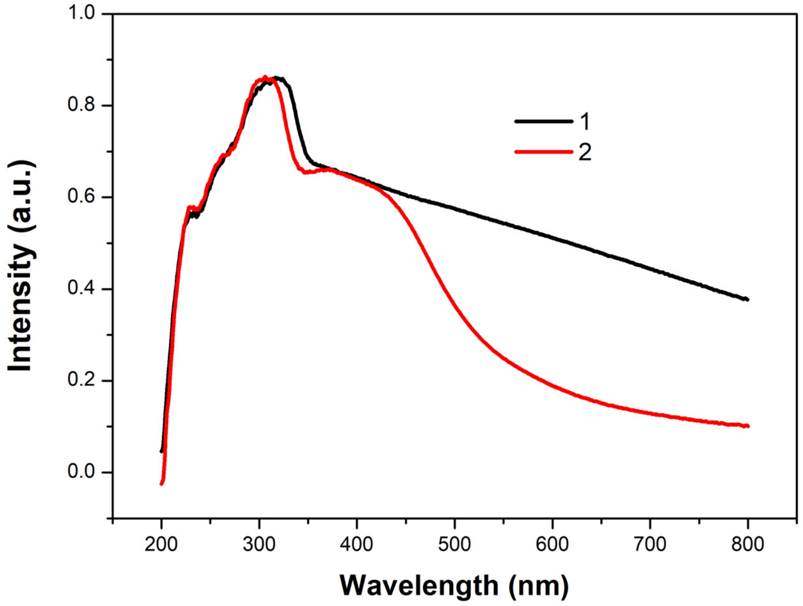

Figure S4 Solid-state UV-Vis spectra for 1 and 2.

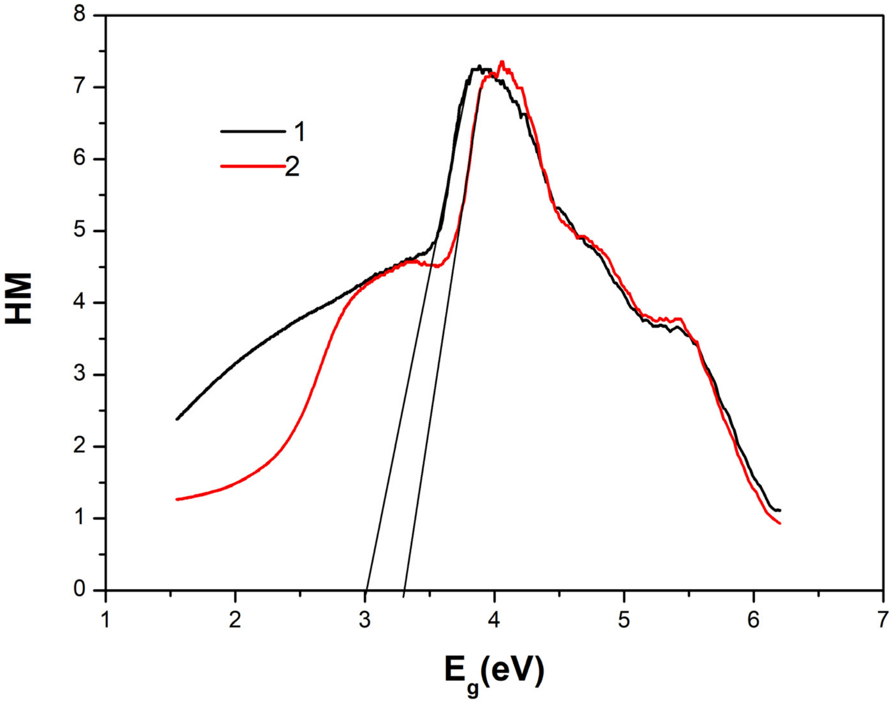

Figure S5. The optical band gap plots for 1 and 2.

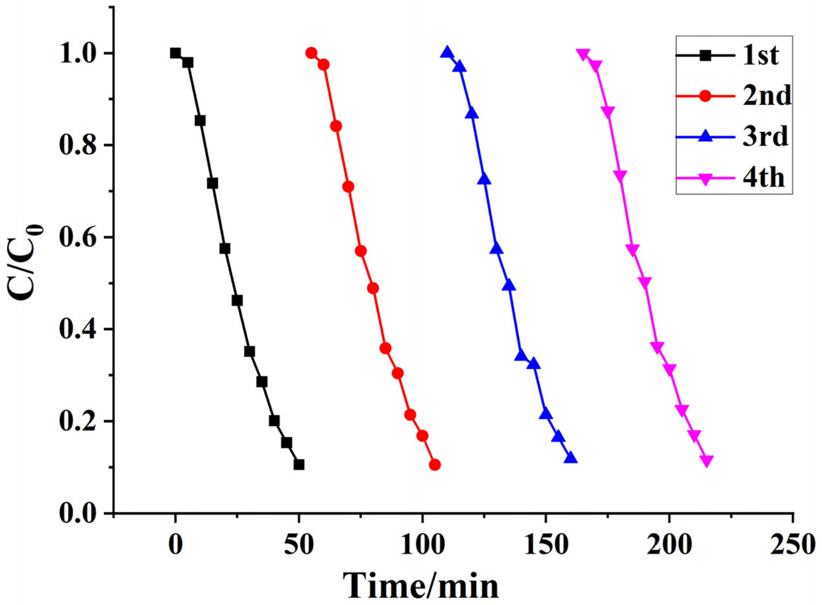

Figure S6. The photocatalytic recycling experiments for the photodegradation of PNP using **1.**

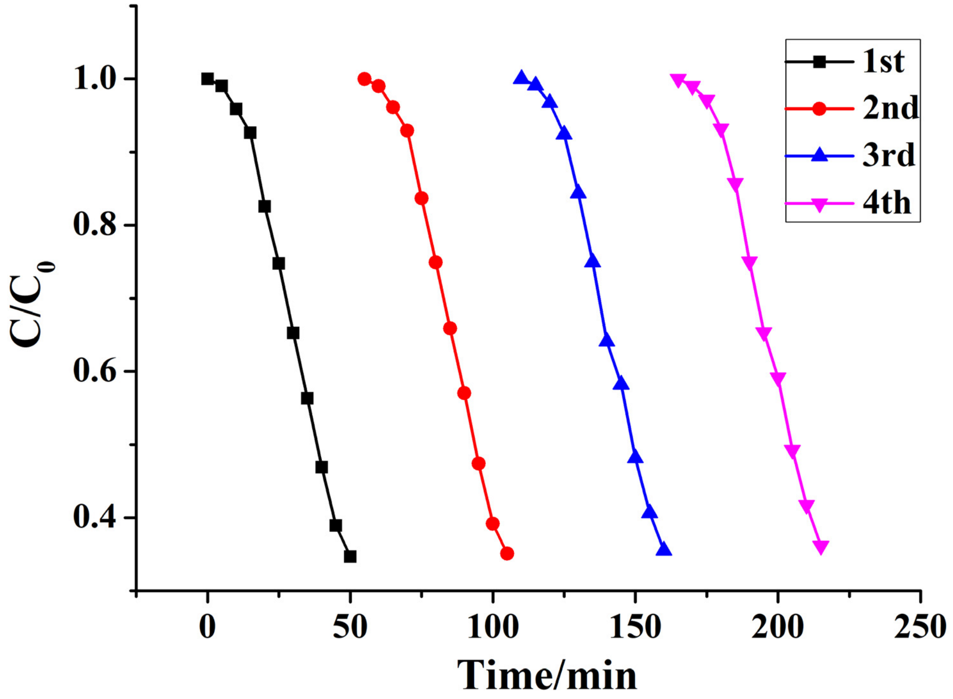

Figure S7. The photocatalytic recycling experiments for the photodegradation of PNP using **2.**

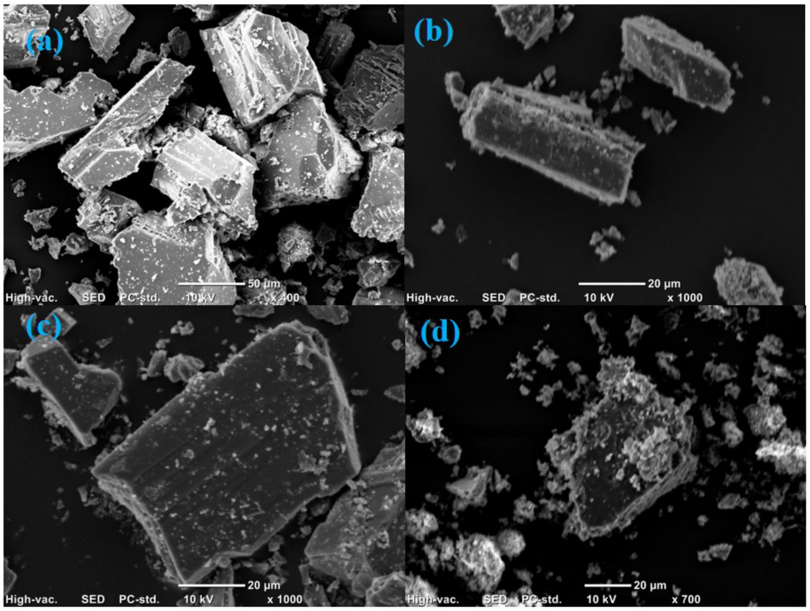

Figure S8. SEM images for **1** (a) before catalysis and (b) after catalysis and SEM images for **2** (c) before catalysis and (d) after catalysis.

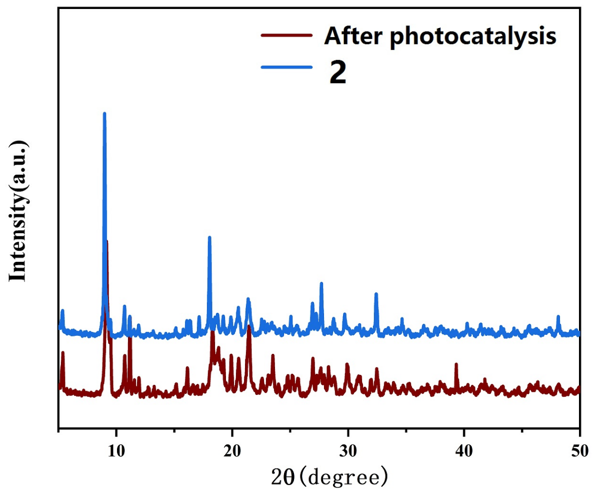

Figure S9. The PXRD plots for **2** after photocatalysis.

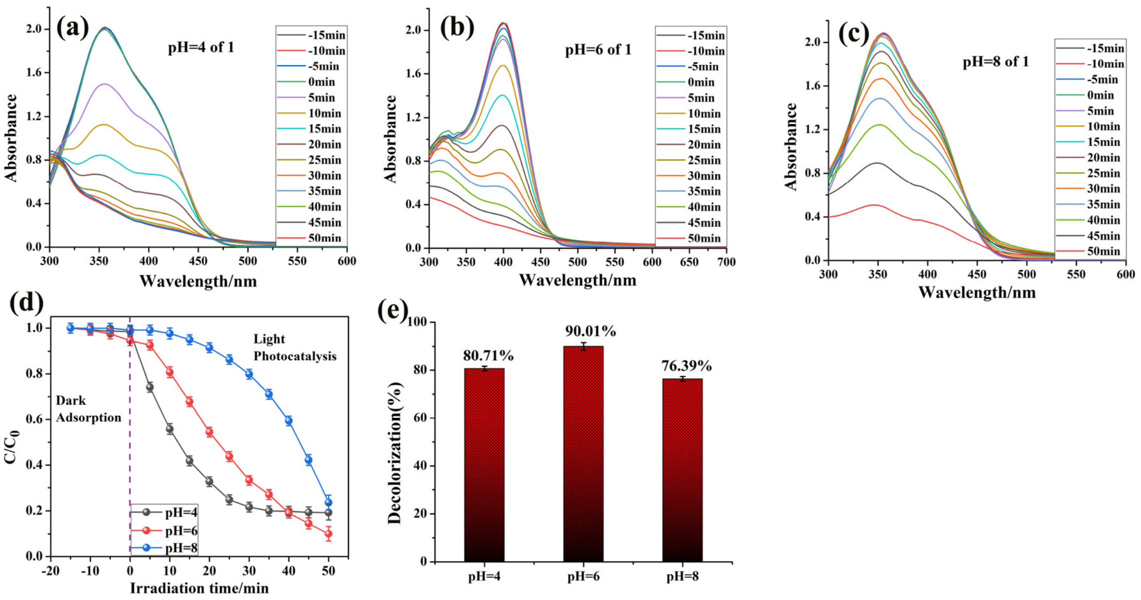

Figure S10. (a)–(c) The photocatalytic efficacy under various pH values; (d) the relationship of the line for C_0/C and t under different pH values; (e) the comparison efficiency under various pH values.

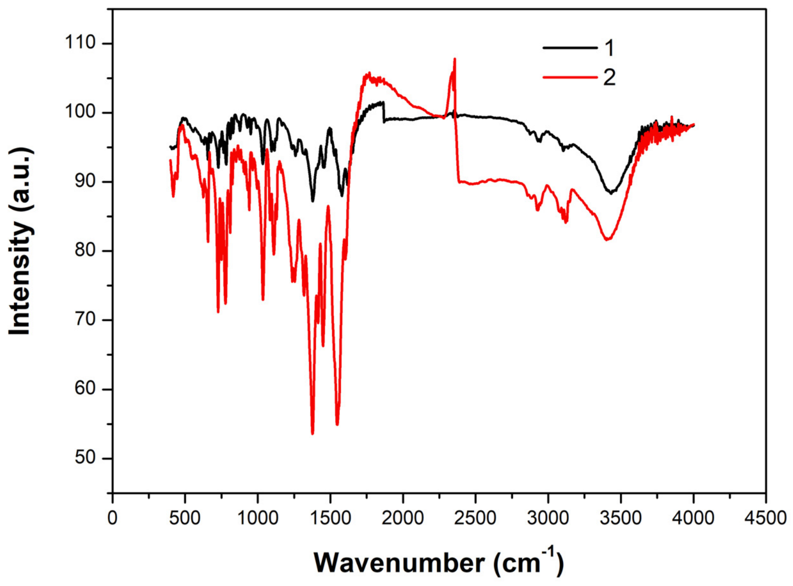

Figure S11. view of the IR for MOFs **1** and **2.**

The textural properties of MOFs **1** and **2** were also explored using the N_2 adsorption method at 77 K (Fig. S11). It was observed that isotherms with a continuous increase at low relative pressure (P/P_0 < 0.05) and a small hysteresis loop were to be found at high relative pressure (0.45 < P/P_0 < 1), which indicates that micropore formations existed. While the MOF **1** had the higher BET surface area and pore volume among the remaining products, MOF **2** showed the lower value, which may be caused by the dense packing of the structure dimension. The DFT pore size distribution of all the materials was smaller than 0.50 nm (insert).

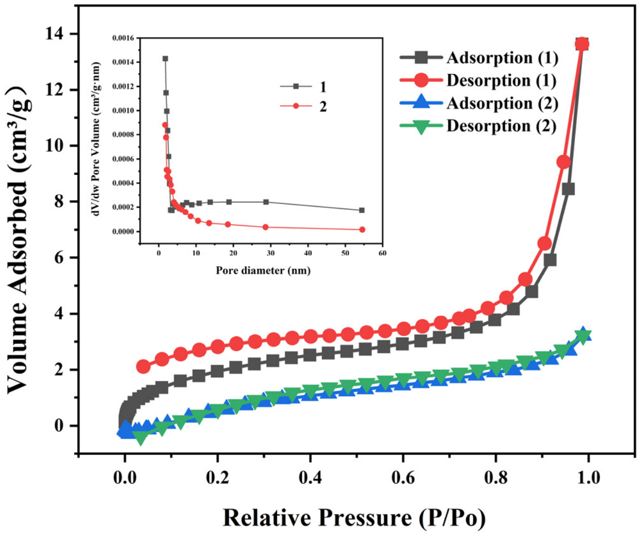

Figure S12. Adsorption–desorption isotherms of the as-synthesized samples in this work (insert: pore size distribution curves).

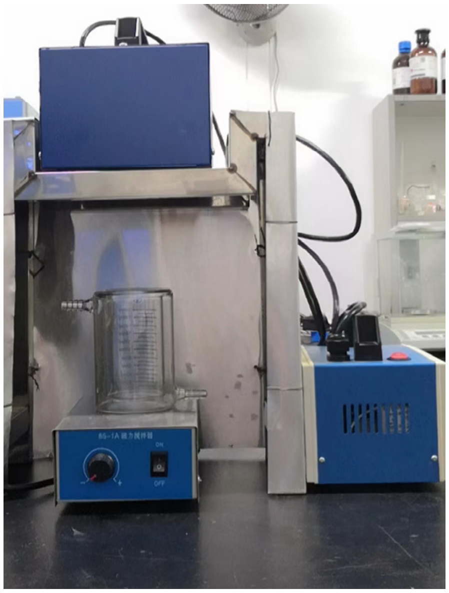

Figure S13. Photograph of the photocatalytic setup in this system.

Table S1. Crystallographic data and structure refinement details for 1-2

Parameter	**1**	**2**
Formula	$C_{34}H_{30}N_4O_{11}Zn_2$	$C_{34}H_{28}Cd_2N_4O_{10}$
Formula weight	801.36	877.40
Crystal system	Triclinic	Triclinic
Space group	*P-1*	*P-1*
Crystal Color	Colorless	Yellow
a, Å	9.2800(8)	9.7823(9)
b, Å	13.5954(11)	10.2446(9)
c, Å	14.1304(11)	17.1486(15)
α, °	83.816(1)	102.586(1)
β, °	76.113(1)	94.231(2)
γ, °	74.618(1)	105.858(1)
V, Å^3	1666.8(2)	1597.1(2)
Z	2	2
ρ_{calcd}, g/cm^3	1.597	1.825
μ, mm^{-1}	1.508	1.399
F(000)	820	872
θ Range, deg	1.5,27.7	1.2,27.6
Reflection collected	10175	7004
Independent reflections (R_{int})	0.023	0.019
Reflections with $I > 2\sigma(I)$	5164	5594
Number of parameters	471	451
R_1, wR_2 ($I > 2\sigma(I)$)*	0.0409, 0.1111	0.0344,0.0972
R_1, wR_2 (all data)**	0.0697, 0.1407	0.0499,0.0820

* $R = \sum(F_o - F_c)/\sum(F_o)$, ** $wR_2 = \{\sum[w(F_o^2 - F_c^2)^2]/\sum(F_o^2)^2\}^{1/2}$.

Table S2. Selected bond distances (Å) and angles (deg.) for **1-2**

	**1**		
Zn(1)-O(1)	1.928(3)	Zn(1) -N(1)	1.986(3)
Zn(1) -O(8)#1	2.022(3)	Zn(1) -O(10)#2	1.925(3)
Zn(2) -O(3)	1.945(3)	Zn(2)-O(4)	2.480(3)
Zn(2)-O(11)	2.015(3)	Zn(2)-N(3)	1.993(4)
Zn(2)-O(7)#3	1.981(3)		
	**2**		
Cd(1)-O(1)	2.213(4)	Cd(1)-O(3)#1	2.378(3)
Cd(1)-N(1)	2.262(4)	Cd(1)-O(4)#1	2.345(3)
Cd(1)-O(9)#2	2.344(3)	Cd(1)-O(7)#1	2.428(3)
Cd(2)-O(7)	2.559(3)	Cd(2)-O(8)	2.270(3)
Cd(2)-N(3)	2.223(4)	Cd(2)-O(9)#3	2.323(3)
Cd(2)-O(10)#3	2.672(3)	Cd(2)-O(2)#4	2.401(3)
Cd(2)-O(10)#5	2.344(3)		
	**1**		
O(1) -Zn(1) -N(1)	113.61(11)	O(1)-Zn(1)-O(8)#1	103.75(11)
O(1)-Zn(1)-O(10)#2	129.34(12)	O(8)#1-Zn(1)-N(1)	102.73(11)
O(10)#2-Zn(1)-N(1)	108.86(12)	O(8)#1-Zn(1)-O(10)#2	92.30(11)
O(3)-Zn(2) -O(4)	57.92(10)	O(3)-Zn(2)-O(11)	117.25(11)
O(3)-Zn(1) -N(3)	126.56(13)	O(3)-Zn(2)-O(7)#3	100.76(11)
O(4)-Zn(2)-O(11)	92.65(10)	O(4)-Zn(2)-N(3)	90.85(12)
O(4)-Zn(2)-O(7)#3	157.20(10)	O(11)-Zn(2)-N(3)	105.20(14)
O(7)#3-Zn(2)-O(11)	90.69(11)	O(7)-Zn(2)-N(3)	110.00(12)
	**2**		
O(1)-Cd(1)-N(1)	101.40(13)	O(1)-Cd(1)-O(9)#2	116.31(11)
O(1)-Cd(1)-O(3)#1	92.02(11)	O(1)-Cd(1)-O(4)#1	145.34(12)
O(1)-Cd(1)-O(7)#1	81.33(11)	O(1)-Cd(1)-C(8)#1	118.87(12)
O(9)#2-Cd(1)-N(1)	95.36(12)	O(3)#1-Cd(1)-N(1)	97.95(12)
O(4)#1-Cd(1)-N(1)	95.83(13)	O(7)#1-Cd(1)-N(1)	170.00(12)
N(1)-Cd(1)-C(8)#1	98.27(13)	O(3)#1-Cd(1)-O(9)#2	145.49(10)
O(4)#1-Cd(1)-O(9)#2	91.45(11)	O(7)#1-Cd(1)-O(9)#2	74.92(10)
O(9)#2-Cd(1)-C(8)#1	118.61(11)	O(3)#1-Cd(1)-O(4)#1	55.66(10)
O(3)#1-Cd(1)-O(7)#1	91.53(10)	O(3)#1-Cd(1)-C(8)#1	27.81(11)
O(4)#1-Cd(1)-O(7)#1	86.88(10)	O(4)#1-Cd(1)-C(8)#1	27.86(11)
O(7)#1-Cd(1)-C(8)#1	88.63(10)	O(7)-Cd(2)-O(8)	54.26(10)
O(7)-Cd(2)-N(3)	171.21(12)	O(7)-Cd(2)-C(23)	27.46(11)
O(7)-Cd(2)-O(9)#3	72.82(10)	O(7)-Cd(2)-O(10)#3	107.40(9)
O(2)#4-Cd(2)-O(7)	83.98(10)	O(7)-Cd(2)-O(10)#5	85.53(10)

Symmetry Codes: **For 1**: #1=x, y, 1+z; #2= -x, 2-y, 1-z; #3=1-x, 1-y, -z; **For 2**: #1= x, 1+y, z;

#2= -1+x, 1+y, z; #3= -1+x, y, z; #4= x, -1+y, z; #5=2-x, 1-y, 1-z.

Table S3. Hydrogen bond distances (Å) and angles (deg.) for **1-2**

Contact D-H···A	Distance, Å			Angle D-H···A, deg
	D-H	H···A	D···A	
**1**				
O(11)-H(11A)···O(2)	0.8600	1.8800	2.630(5)	145.00
O(11)-H(11B)···O(9)	0.8600	1.8400	2.660(4)	159.00
C(5)-H(5)···O(3)	0.9300	2.4200	2.746(5)	100.00
C(14)-H(14)···O(10)	0.9300	2.5700	3.388(5)	146.00
C(16)-H(16A)···O(5)	0.9700	2.5500	3.187(5)	123.00
C(25)-H(25)···O(3)	0.9300	2.5100	3.412(5)	163.00
**2**				
C(9)-H(9A)···O(6)	0.9700	2.4500	3.140(6)	128.00
C(9)-H(9B)···O(2)	0.9700	2.4500	3.376(6)	159.00
C(11)-H(11)···O(5)	0.9300	2.5100	2.854(6)	102.00
C(18)-H(18)···O(3)	0.9300	2.4500	3.326(5)	158.00
C(22)-H(22)···O(4)	0.9300	2.4100	3.339(5)	173.00
C(27)-H(27)···O(3)	0.9300	2.3800	3.307(6)	171.00
C(30)-H(30)···O(7)	0.9300	2.5300	3.429(6)	162.00
